# Cognitive psychology-based artificial intelligence review

**DOI:** 10.3389/fnins.2022.1024316

**Published:** 2022-10-06

**Authors:** Jian Zhao, Mengqing Wu, Liyun Zhou, Xuezhu Wang, Jian Jia

**Affiliations:** ^1^School of Information Science and Technology, Northwest University, Xi’an, China; ^2^Medical Big Data Research Center, Northwest University, Xi’an, China; ^3^School of Mathematics, Northwest University, Xi’an, China

**Keywords:** cognitive psychology, artificial intelligence, cognitive theory, behavioral science, human–computer interaction

## Abstract

Most of the current development of artificial intelligence is based on brain cognition, however, this replication of biology cannot simulate the subjective emotional and mental state changes of human beings. Due to the imperfections of existing artificial intelligence, this manuscript summarizes and clarifies that artificial intelligence system combined with cognitive psychology is the research direction of artificial intelligence. It aims to promote the development of artificial intelligence and give computers human advanced cognitive abilities, so that computers can recognize emotions, understand human feelings, and eventually achieve dialog and empathy with humans and other artificial intelligence. This paper emphasizes the development potential and importance of artificial intelligence to understand, possess and discriminate human mental states, and argues its application value with three typical application examples of human–computer interaction: face attraction, affective computing, and music emotion, which is conducive to the further and higher level of artificial intelligence research.

## Introduction

At present, in the development of artificial intelligence (AI), the scientific community is mostly based on brain cognition research (Nadji-Tehrani and Eslami, 2020), which is to reproduce the real physiological activities of our human brain through computer software. This replication of the biology of the human brain cannot well simulate the subjective psychological changes (Zador, 2019). For example, in terms of memory, human memory forgetting is non-active, and the more we want to forget the more memorable it becomes, while machine forgetting is an active deletion, which deviates from our psychological expectations. In the process of promoting the progress of artificial intelligence, psychology and its derived philosophy of mind play an important role directly or indirectly, can be considered as one of the fundamental supporting theories of AI. For example: The current reinforcement learning theory in AI is inspired by the behaviorist theory in psychology, i.e., how an organism gradually develops expectations of stimuli in response to rewarding or punishing stimuli given by the environment, resulting in habitual behavior that yields maximum benefit. The current challenges faced by the artificial intelligence community – the emotional response of artificial intelligence machines, decision making in ambiguous states also need to rely on breakthroughs in the corresponding fields of psychology. Psychology and its derived philosophy of mind can be considered as one of the fundamental support theories for artificial intelligence (Miller, 2019). Cognitive psychology is mainly a psychological science that studies the advanced mental processes of human cognition, including the degree of thinking, deciding, reasoning, motivation and emotion. The most important feature that distinguishes humans from machines is that humans process external input by feeding back different attitudes toward things through our already internalized knowledge units about the external world, stimulating different subjective emotional orientations such as satisfaction, dissatisfaction, love, dislike and so on. These labeled emotional traits are generated by human cognitive psychology. By measuring subjective emotional changes, the internal knowledge structure is updated and the artificial intelligence machine is guided to re-learn, so that human attitudes, preferences and other subjective emotional experiences are given in AI (Kriegeskorte and Douglas, 2018; Pradhan et al., 2020).

Research on artificial intelligence is still in the developmental stage in terms of simulating human memory, attention, perception, knowledge representation, emotions, intentions, desires, and other aspects (Shi and Li, 2018). As the existing AI is not perfect, the AI system combined with cognitive psychology is the research direction of AI: Promote the development of artificial intelligence, endow the computer with the ability to simulate the advanced cognition of human beings, and carry out learning and thinking, so that computers can recognize emotions, understand human feelings, and finally achieve dialog and empathy with humans and other AI.

In terms of existing research results and methods, artificial intelligence combines new theories and methods such as psychology, brain science and computer science to conduct artificial intelligence machine simulation on people’s psychological activities, reproduce people’s psychology, integrate and promote each other, and jointly create more universal and autonomous artificial intelligence, which can better realize human–computer interaction (Yang et al., 2018) and further improve the level of social intelligence. At the same time, with the development of psychology, the scope of research and the choice of research objects are more extensive and universal, making artificial intelligence products have the conditions for rapid penetration into the field of psychology, resulting in research products such as facial expression-based emotion recognition system, public opinion analysis based on big data analysis technology, intelligent medical image grading or diagnosis, suicide early warning system and intelligent surveillance management system, which in turn promotes the development of psychology and shortens the research cycle of psychology (Branch, 2019).

The review of artificial intelligence based on cognitive psychology at this stage is not comprehensive enough. This manuscript does the following: (a) introduce the current situation and progress of artificial intelligence research on cognitive psychology in recent years; (b) analyze the experimental data on the application examples of cognitive psychology in artificial intelligence; (c) summarize and outlook the related development trend.

## Research status

Research related to artificial intelligence in cognitive psychology is trending in recent years. In the mid-1980s, the term “Kansei Engineeirng” was introduced in the Japanese science and technology community (Ali et al., 2020). They interpret sensibility as human psychological characteristics, study people’s perceptual needs with engineering methods, and then conduct in-depth research on people’s perceptual information, and the scope of their research is the human psychological perceptual activities.

Professor Wang Zhiliang of University of Science and Technology Beijing proposed the concept of “artificial psychology” on this basis: The artificial psychological theory is to use the method of information science to realize the more comprehensive content of people’s psychological activities. He broadened the range of psychological characteristics involved in “Kansei Engineeirng,” including low-level psychological activities and high-level processes of psychological activities. It is the reflection of human brain on objective reality, which makes artificial psychology have a new meaning and broader content.

Minsky, one of the founders of artificial intelligence, proposed the theory of “society of mind” in his 1985 monograph “The Society of Mind” (Auxier, 2006), which attempts to combine the approaches of developmental psychology, dynamic psychology and cognitive psychology with the ideas of artificial intelligence and computational theory. Since then, the research on endowing the computer with emotional ability and enabling the computer to understand and express emotions has set off an upsurge in the computer field.

In 1978, deepmind team put forward the theory of mind (Rabinowitz et al., 2018). In a broad sense, it refers to the ability of human beings to understand the psychological state of themselves and others, including expectations, beliefs and intentions, and to predict and explain other people’s behaviors based on this. In 2017, in the case study of deepmind team, the research team selected “shape preference” as the entry point for detecting neural networks. It found that, like human beings, the network’s perception of shape exceeded its preference for color and material, which proved that neural networks also have “shape preference” (Ritter et al., 2017). In 2018, the Deepmind team open sourced the simulation psychology laboratory Psychlab, which uses knowledge in cognitive psychology and other fields to study the behavior of artificial agents in controlled environments, thereby simulating human behavior (Leibo et al., 2018).

**FIGURE 1:**
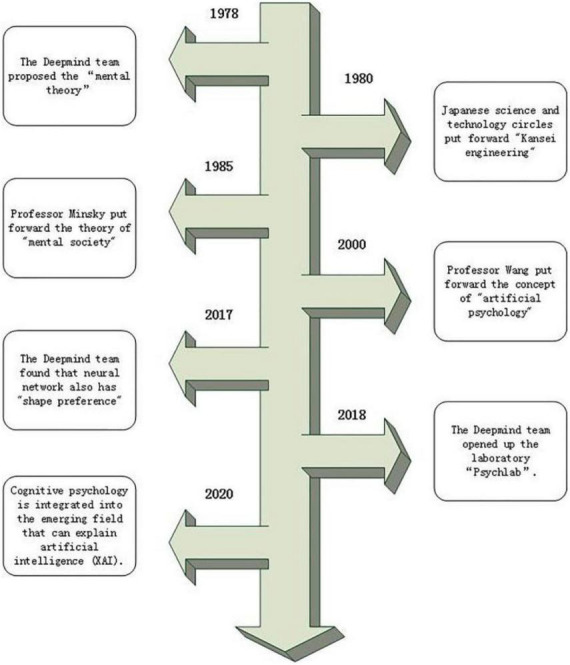
The evolution of artificial intelligence in cognitive psychology.

In 2020, Taylor incorporated cognitive psychology into the emerging field of explainable artificial intelligence (XAI) with the aim of improving the interpretability, fairness, and transparency of machine learning. Figure 1 shows the evolution of AI in cognitive psychology (Taylor and Taylor, 2021).

## Example of cognitive psychological artificial intelligence applications

Cognitive psychology has been very instructive for the development of AI, and current AI design makes extensive reference to human cognitive models. The process of human mental activity is simulated in various aspects such as attention, encoding, and memory. Cognitive psychological artificial intelligence has been researched in many fields. In this manuscript, we study the basic contents and latest progress of psychology and brain science, and systematically analyze and summarize three typical application scenarios: face attraction, affective computing, and music emotion. These examples guide the learning of AI through the higher mental processes of human cognition, including subjective mental orientations such as thinking and emotion. Artificial intelligence is trained to recognize emotions, understand human feelings, and replicate the human psyche, which in turn accelerates research in cognitive psychology.

### Face attraction

Different aesthetic judgments of human faces are one of the most common manifestations of human visual psychology, which is an important source of social emotion generation and plays a role in human social interaction and communication (Han et al., 2020). In daily life, most people think that beauty is a subjective feeling, however, scientists have broken the long-held belief that beauty lacks objectivity and found a high degree of consistency in human perception of facial beauty across race, age, gender, social class, and cultural background. This observation also suggests that face attractiveness reflects to some extent general human psychological commonalities.

SCUT-FBP5500, a database for face attractiveness prediction, was collected and released by the Human–Computer Interaction Laboratory of South China University of Technology. The dataset has 5,500 face frontal photos with different attributes (male/female, age and so on) and different feature labels including face feature point coordinates, face value score (1∼5), face value score distribution and so on. These mental preference features were experimentally used as training data to form mental state embeddings. Then different computer models (AlexNet, ResNet-18, ResNeXt-50) were used for classification, regression and ranking to form a deep learning-based face attractiveness template (Huang, 2017). Evaluate the benchmark according to various measurement indicators, including Pearson correlation coefficient (PC), maximum absolute error (MAE) and root mean square error (RMSE) evaluation model. We used the five-fold method to analyze the performance of the face attractiveness templates under different computer models, and found that the Pearson correlation coefficient was above 0.85, the maximum absolute error was around 0.25, and the root mean square error was between 0.3 and 0.4 (Liang et al., 2018).

Elham Vahdati proposes and evaluates a face facial attractiveness prediction method using facial parts as well as a multi-task learning scheme. First, face attractiveness prediction is performed using a deep convolutional neural network (CNN) pre-trained on a massive face dataset to automatically learn advanced face representations. Next, the deep model is extended to other facial attribute recognition tasks using a multi-task learning scheme to learn the best shared features for three related tasks (such as facial beauty assessment, gender recognition, and race recognition). To further improve the accuracy of the attractiveness computation, specific regions of the face image (such as left eye, nose, and mouth) as well as the entire face are fed into a multi-stream CNN (such as three dual-stream networks). Each dual-stream network uses partial features of the face and the full face as input. Extensive experiments were conducted on the SCUT-FBP5500 benchmark dataset, with a significant improvement in accuracy (Vahdati and Suen, 2021).

Irina Lebedeva, Fangli Ying learned a large number of aesthetic preferences shared by many people during the meta-training process. The model is then used on new individuals with a small sample of rated images in the meta-testing phase. These experiments were conducted on a facial beauty dataset that included faces of different races, genders, and age groups and were scored by hundreds of volunteers with different social and cultural backgrounds. The results show that the proposed method is effective in learning individual beauty preferences from a limited number of annotated images and outperforms existing techniques for predicting facial beauty in terms of quantitative comparisons (Lebedeva et al., 2022).

We summarize the theoretical concepts of artificial intelligence based on cognitive psychology, and do relevant research on this basis. Since the database of face attractiveness needs to be characterized by large samples, diversity and universality, in 2016, we built a Chinese face database containing different ethnicities of different genders. In 2017, considering that the contour structure, geometric features and texture features of faces change with age, in order to study the impact of different face features on the evaluation of face attractiveness under different age groups, we built a middle-aged and elderly face database. In 2018, we used migration learning to migrate the face feature point templates of face recognition to the construction of face attractiveness face templates, and constructed a geometric feature-based face attractiveness evaluation model. In 2019, we established a face database of Chinese males in different eras, and studied the aesthetic characteristics and trends of Chinese males from the perspective of era development. An 81-point face feature point template for face attractiveness analysis was also proposed through feature vector analysis of face image quantification and light model. In 2020, a comprehensive facial attractiveness evaluation system was proposed considering the combined effects of face structure features, facial structure features, and skin texture features on face attractiveness scores, and the experimental results are shown in Table 1, when these three features are integrated with each other, the Pearson correlation coefficient reached the highest value of 0.806 (Zhao et al., 2019a,b, c; Zhao et al., 2020).

**TABLE 1:** Performance of face attractiveness prediction with different features.

Predictive performance	F	S	LBP	F × S	F × LBP	S × LBP	F × S × LBP
LR	0.502	0.616	0.658	0.683	0.654	0.637	0.722
KNN	0.619	0.672	0.694	0.753	0.771	0.782	0.794
SVM-LIN	0.649	0.738	0.712	0.768	0.732	0.724	0.797
SVM-RBF	0.702	0.713	0.741	0.763	0.754	0.781	0.806

Through years of research at the intersection of artificial intelligence + face attractiveness, it is shown that although it may be difficult to establish a clear, interpretable and accepted set of rules to define face attractiveness. However, it is possible to explore the relationship between ordinary faces and attractive faces, and the qualitative study of face aesthetic preferences can be described quantitatively by artificial intelligence. The results highly fit contemporary aesthetic standards, demonstrating that it is feasible for computers to simulate advanced human cognitive abilities to recognize emotions and understand human feelings, and that the development of artificial intelligence based on cognitive psychology has potential and significance.

### Affective computing

Emotion is a psychological state of positive or negative attitude toward external things and objective reality, and can be defined as a group of psychological phenomena expressed in the form of emotions, feelings or passions. Emotions not only refer to human emotions, but also refer to all human sensory, physical, psychological and spiritual feelings. Damasio found in his research that due to the defect of the channel between the cerebral cortex (Cortex: control of logical reasoning) and the limbic system (Limbic System: control of emotion), his “patients” despite having normal or even supernormal rational thinking and logical reasoning. However, their decision-making ability has encountered serious obstacles (Bechara et al., 2000), proving that human intelligence is not only manifested in normal rational thinking and logical reasoning abilities, but also in rich emotional abilities.

More than 40 years ago, Nobel Laureate Herbert Simon emphasized in cognitive psychology that problem solving should incorporate the influence of emotions (Simon, 1987). As one of the founders of artificial intelligence, Professor Marvin Minsky of the Massachusetts Institute of technology of the United States first proposed the ability to make computers have emotion. In his monograph the society of mind, he emphasized that emotion is an indispensable and important ability for machines to achieve intelligence. The concept of affective computing was first introduced by Picard (1995), when she stated that “affective computing is computing that can measure and analyze and influence emotions in response to human outward expressions” (Picard, 2003). This opened up a new field of computer science, with the idea that computers should have emotions and be able to recognize and express them as humans do, thus making human–computer interaction more natural.

As an important means of interpersonal communication, emotion conveys the information of emotional state and explains complex psychological activities and behavioral motives through physiological indicators such as human language text, intonation volume change, facial expression, action posture and brain wave.

In, Ekman (1972) an American professor of psychology, proposed a method for the expression of facial emotions (Facial Motor Coding System FACS) (Buhari et al., 2020). By the combination of different coding and motor units, complex expression changes can be formed on the face. Facial motion coding system FACS can analyze emotions using deep region and multi-label learning (DRML) architecture, using feedforward functions to induce important facial regions, and able to learn weights to capture structural information of the face. The resulting network is end-to-end trainable and converges faster than alternative models with better learning of AU relationships (Zhao et al., 2016). The corresponding emotion computation formula can be derived based on the facial motion encoding, as Table 2 shown.

**TABLE 2:** Emotion formula.

Expression	Formula of AU
Happiness	AU6 + AU12
Sadness	AU1 + AU4 + AU15
Surprise	AU1 + AU2 + AU5 + AU26
Fear	AU1 + AU2 + AU4 + AU5 + AU7 + AU20 + AU26
Anger	AU4 + AU5 + AU7 + AU23
Disgust	AU9 + AU15 + AU16
Contempt	AU12 + AU14

In the process of human information interaction, speech is the most common way for people to communicate. As the most basic audiovisual signal, speech cannot only identify different vocalists, but also effectively distinguish different emotional states. International research on emotional speech focuses on the analysis of acoustic features of emotions, such as rhythm, sound source, resonance peaks and spectrum and so on (Albanie et al., 2018). In recent years, deep learning has been widely studied and has many applications in speech emotion computation. Dongdong Li proposed a bidirectional long short-term memory network with directed self-attention (BLSTM-DSA). Long Short Term Memory (LSTM) neural networks can learn long-term dependencies from learned local features. In addition, Bi-directional Long Short-Term Memory(Bi-LSTM) can make the structure more robust through the direction mechanism, and the direction analysis can better identify the hidden emotions in sentences. Also, the autocorrelation of speech frames can be used to deal with the problem of missing information, thus introducing a self-attention mechanism in Speech Emotion Recognition (SER). When evaluated on the Interactive Emotional Binary Motion Capture (IEMOCAP) database and the Berlin Emotional Speech Database (EMO-DB), BLSTM-DSA achieves a recognition rate of over 70% for each algorithm on the speech emotion recognition task (Li et al., 2021).

Human posture often carries emotional information during interaction. Researchers have combined human posture with artificial intelligence to quantitatively assess the external representation of a person’s mental state in the face of different situations through a series of movement and body information capture devices. For example, the intelligent seat is applied to the driver’s seat of the vehicle to dynamically monitor the emotional state of the driver and give timely warnings. Some scientists in Italy also conduct automatic emotional analysis on office staff through a series of posture analysis to design a more comfortable office environment.

Electroencephalographic(EEG) is a graph obtained by amplifying and recording the spontaneous biological potential of the brain from the scalp through precise electronic instruments. It has been widely used in the field of emotion recognition. The DEAP dataset used to study human emotional states (Luo et al., 2020), recording EEG and peripheral physiological signals from 32 participants watching 40 one-minute long music video clips. Participants rated each video according to arousal, potency, like/dislike, dominance, and familiarity. Correlations between EEG signal frequencies and participants’ ratings were investigated by emotional label retrieval, and decision fusion was performed on classification results from different modalities. The experiments obtained an average recognition rate of up to 84.2% and up to 98% by identifying a single emotional state, while for two, three and four emotions, the average recognition rate was up to 90.2, 84.2, and 80.9%, respectively. Table 3 shows the validated classification accuracy of the DEAP dataset based on different recognition models (Khateeb et al., 2021).

**TABLE 3:** Classification accuracy of deap dataset based on different recognition models.

Stimulus	Classifier	Emotions	Subjects	Accuracy
Video	GELM	4	32	69.67
Audio	MLP	4	30	78.11
Video	Nearest neighbour	4	32	73.62
Video	Domain-adaptation	5	14	39.05
Video	SVM	Valence-dominance	10	63.04
Video	K-NN	2	30	69.50

**FIGURE 2:**
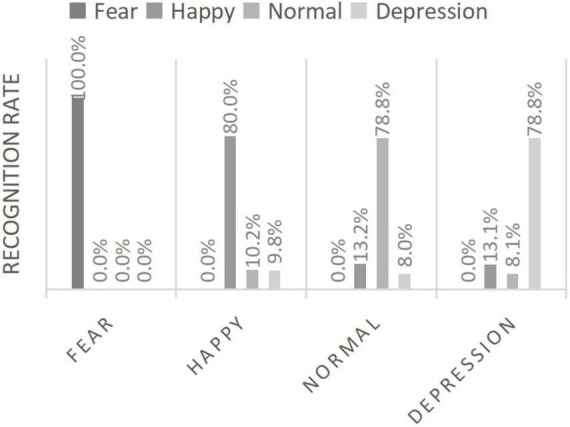
Speech emotion recognition rate.

**FIGURE 3:**
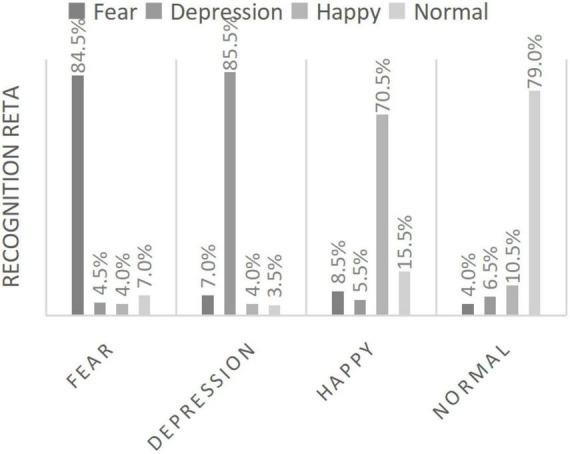
Face facial emotion recognition rate.

Our research group has also carried out relevant research on multimodal affective computing, and has a patent for automatic diagnosis of depression based on speech and facial expression: By combining facial gesture features, we propose a new double dictionary idea with gesture robustness. In 2016, feature extraction and evaluation of depressed speech were performed, and in the following year, we proposed to use the change of expression of depressed patients as one of the evaluation indicators to determine whether they suffer from depression as well. Figures 2 and 3 shows the data.

In 2018, a new automatic depression assistant discrimination algorithm integrating speech and facial expression was proposed. Firstly, the signal enhancement was performed for depressed speech; the fundamental frequency and the first three resonance peaks features were extracted by the inverse spectral method, and the energy, short-time average amplitude and Mel-Frequency Ceptral Coefficients(MFCC) features were extracted; the speech recognition model and the facial expression recognition model were established to assist in judging whether a person has depression; finally, the Adaboost algorithm based on back propagation(BP) neural network was proposed and validated in a practical situation for an automatic depression-assisted detection system. As Table 4 shown, the recognition rate of the depression detection algorithm based on fused speech and facial emotion reached 81.14%. The development of artificial intelligence provides a more objective judgment basis for the diagnosis of depression in psychological medical health, which has cutting-edge and application value (Zhao et al., 2019d).

**TABLE 4:** The integration of voice and facial expression recognition rate.

	Speech recognition results (%)	Facial expression recognition results (%)	After fusion recognition results (%)
Before the speech signal enhancement	62.4	85.5	75.75
Enhanced speech signal	78.8	85.5	82.29

Affective computing is a combination of computational science with physiology science, psychological science, cognitive science and other disciplines. Based on the common cognition and knowledge structure of human on different emotional expressions, it studies the emotions in the process of human-human interaction and human–computer interaction, and guides the design of artificial intelligence with emotion recognition and feedback functions, understands human emotional intentions and makes appropriate responses to achieve human–computer emotional interaction.

### Music emotion

Extensive research on musical emotions suggests that music can trigger emotional activity in listeners. Scientists believe that when a person is in a beautiful and pleasant musical environment, the body secretes an active substance that is beneficial to health and helps eliminate psychological factors that cause tension, anxiety, depression and other adverse psychological states (Rahman et al., 2021). People’s preference for different kinds of music is not without rules, after psychological cognition and data test, there is a precise music signal α value can measure the ear-pleasant degree. The closer the music signal α is to the value 1, the better it sounds. The value of α also can be obtained by artificial intelligence (Banerjee et al., 2016). This shows that people’s psychological state toward music can be judged by machines, and further research can be based on this law to simulate good-sounding music in line with public aesthetics and realize the interaction between emotions and machines.

**FIGURE 4:**
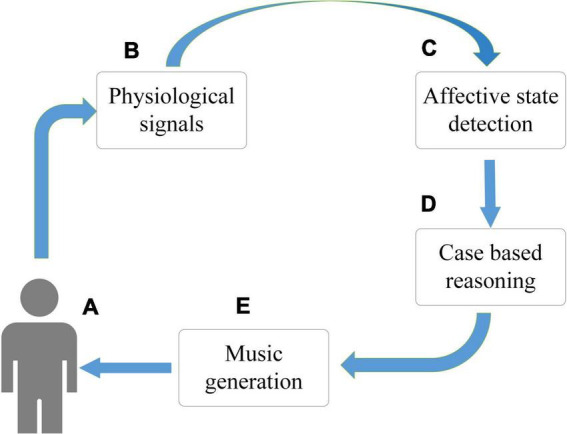
The proposed affective brain-computer music interface (aBCMI). The system consists of five key elements: **(A)**. The user of the system **(B)**. The user’s physiological signal acquisition module (including the electroencephalogram (EEG), electrocardiogram (ECG) and respiration rate) **(C)**. An emotional state detection system for identifying a current emotional state that a user is experiencing **(D)**. A case-based reasoning system that determines how a user moves from his current emotional state to a new target emotional state **(E)**. The music generator is used to play music for the user. The case-based reasoning system identifies the most appropriate emotional trajectory and moves them to the target emotional state.

As Figure 4, a team of researchers from the University of Reading and the University of Plymouth in the UK developed and evaluated an affective brain-computer music interface (aBCMI) for detecting a user’s current emotional state and attempting to modulate it by playing music generated by a music composition system based on specific emotional goals.

The affective state detection method achieved statistically significant online single-trial classification accuracy in classifying user potency in seven-eighths of participants and in classifying user arousal in three-eighths of participants. The mean accuracy for affective state detection was 53.96% (chemotaxis) and 53.80% (arousal) (Daly et al., 2016). The experimental data also demonstrate that the aBCMI system is able to detect the emotional states of most of the participants and generate music based on their emotional states to achieve “happy” and “calm” mental states. By visualizing abstract mental states, extracting features from changes in emotional states, and quantifying different emotions in different musical environments, the aBCMI system can effectively characterize and provide feedback to regulate current emotional states, realizing the combination of psychology and artificial intelligence.

Musical emotion regulation aims to record physiological indicators from users with a signal acquisition component in order to capture the cognitive and physiological processes associated with their current affective state. Features are extracted from the physiological signals that most likely correspond to changes in the user’s affective state. Then the case-based reasoning system is used to determine the best method to transfer them to the target emotional state, so as to move the user to the target emotional state.

Dapeng Li and Xiaoguang Liu have also combined incremental music teaching methods to assist therapy. The combination of contextual teaching and artificial intelligence attention theory makes the assisted treatment system more targeted. The design of treatment content more fully takes into account the patient’s actual situation. When designing the music teaching-assisted treatment context, the physician will fully consider various factors of the patient, from the perspective of mobilizing the patient’s interest in the music learning work, to achieve the full activity of brain neurons and more fully access the pathological information around the lesion to promote autoimmunity and subsequent treatment (Li and Liu, 2022).

The evocation of musical emotions is based on functional connections between sensory, emotional and cognitive areas of the brain, including subcortical reward networks common to humans and other animals, such as the nucleus accumbens, amygdala and dopaminergic systems, as well as the evolutionary end of the cerebral cortex with complex cognitive functions. Musical emotions regulate the activity of almost all limbic and paralimbic structures of the brain. Music can induce different emotions, and we can also use music emotions to guide the development of artificial intelligence. Further research is expected in such fields as music generation, education, medical treatment and so on.

## Summary and outlook

Through systematic analysis and application examples, this manuscript points out that the artificial intelligence system combined with cognitive psychology is the development direction of artificial intelligence: to promote the development of artificial intelligence, to give computers the ability to simulate human’s advanced cognition, and to learn and think, so that computers can recognize emotions and understand human feelings, and finally realize dialog and empathy with human beings and other artificial intelligence. Artificial intelligence with human psychological cognition cannot only simulate the rational thinking of “brain,” but also reproduce the perceptual thinking of “heart,” and can realize the emotional interaction between people and machines, machines and machines, similar to human communication.

Nowadays, the theory of artificial intelligence based on cognitive psychology also has imperfections: due to the differences in race, region and growth environment, the evaluation criteria for each subject are not completely consistent, and the random sampling difference is even greater Moreover, mental activities are generally ambiguous and chaotic.

The future interdisciplinary combination of AI and psychology will focus on the following aspects: big data medical, human–computer interaction, brain-computer interface, general artificial intelligence and so on. Through the combination of cognitive science in psychology and AI, breakthroughs in many aspects will be achieved based on multimodal data and extraction of high-dimensional data. The two accomplish each other, complementing each other and developing together.

This manuscript provides a research direction for the development of artificial intelligence to simulate machines with human emotions and to realize human–computer interaction. It has the characteristics of cutting-edge science, which is not only of great theoretical significance, but also has good development potential and application prospects. It is hoped that it can provide research basis for follow-up researchers.

## Author contributions

JZ formulated the research manuscript idea, provided substantial edits to the manuscript and final draft, and aided in the interpretation of the manuscript. MW wrote the main body of the manuscript, participated in revisions, and submitted the final manuscript. LZ contributed to the formulation of the research manuscript idea, provided substantial edits to the manuscript and the final draft, and aided in the interpretation of the manuscript. XW and JJ participated in the conception of the idea and revised the manuscript. All authors contributed to the article and approved the submitted version.
